# Identification of BRCA1-like triple-negative breast cancers by quantitative multiplex-ligation-dependent probe amplification (MLPA) analysis of BRCA1-associated chromosomal regions: a validation study

**DOI:** 10.1186/s12885-016-2848-2

**Published:** 2016-10-19

**Authors:** Eva Gross, Harm van Tinteren, Zhou Li, Sandra Raab, Christina Meul, Stefanie Avril, Nadja Laddach, Michaela Aubele, Corinna Propping, Apostolos Gkazepis, Manfred Schmitt, Alfons Meindl, Petra M. Nederlof, Marion Kiechle, Esther H. Lips

**Affiliations:** 1 Department of Gynecology and Obstetrics, Technische Universität München, Ismaninger Strasse 22, D-81675 Munich, Germany; 2 Biometrics Department, The Netherlands Cancer Institute, Plesmanlaan 121, 1066 CX Amsterdam, The Netherlands; 3 Institute of Pathology, Technische Universität München, Ismaninger Strasse 22, D-81675 Munich, Germany; 4 MRC-Holland, Willem Schoutenstraat 6, 1057 DN Amsterdam, The Netherlands; 5 Helmholtz Zentrum München, Institute of Pathology, Ingolstädter Landstrasse 1, D-85764 Neuherberg, Germany; 6 Department of Pathology, The Netherlands Cancer Institute, Plesmanlaan 121, 1066 CX Amsterdam, The Netherlands; 7 Department of Molecular Pathology, The Netherlands Cancer Institute, Plesmanlaan 121, 1066 CX Amsterdam, The Netherlands; 8 Present address: Department of Pathology, Case Western Reserve University School of Medicine, University Hospitals Case Medical Center, Cleveland, OH USA

**Keywords:** BRCA1, BRCAness, DNA repair, PARP1, MLPA assay, Triple-negative breast cancer

## Abstract

**Background:**

Triple-negative breast cancer (TNBC) with a BRCA1-like molecular signature has been demonstrated to remarkably respond to platinum-based chemotherapy and might be suited for a future treatment with poly(ADP-ribose)polymerase (PARP) inhibitors. In order to rapidly assess this signature we have previously developed a multiplex-ligation-dependent probe amplification (MLPA)-based assay. Here we present an independent validation of this assay to confirm its important clinical impact.

**Methods:**

One-hundred-forty-four TNBC tumor specimens were analysed by the MLPA-based “BRCA1-like” test. Classification into BRCA1-like vs. non-BRCA1-like samples was performed by our formerly established nearest shrunken centroids classifier. Data were subsequently compared with the *BRCA1*-mutation/methylation status of the samples. T-lymphocyte infiltration and expression of the main target of PARP inhibitors, PARP1, were assessed on a subset of samples by immunohistochemistry. Data acquisition and interpretation was performed in a blinded manner.

**Results:**

In the studied TNBC cohort, 63 out of 144 (44 %) tumors were classified into the BRCA1-like category. Among these, the MLPA test correctly predicted 15 out of 18 (83 %) samples with a pathogenic *BRCA1*-mutation and 20 of 22 (91 %) samples exhibiting *BRCA1*-promoter methylation. Five false-negative samples were observed. We identified high lymphocyte infiltration as one possible basis for misclassification. However, two falsely classified *BRCA1*-mutated tumors were also characterized by rather non-BRCA1-associated histopathological features such as borderline ER expression. The BRCA1-like vs. non-BRCA1-like signature was specifically enriched in high-grade (G3) cancers (90 % vs. 58 %, *p* = 0.0004) and was also frequent in tumors with strong (3+) nuclear PARP1 expression (37 % vs. 16 %; *p* = 0.087).

**Conclusions:**

This validation study confirmed the good performance of the initial MLPA assay which might thus serve as a valuable tool to select patients for platinum-based chemotherapy regimens. Moreover, frequent PARP1 upregulation in BRCA1-like tumors may also point to susceptibility to treatment with PARP inhibitors. Limitations are the requirement of high tumor content and high-quality DNA.

## Background

Triple-negative breast cancer (TNBC) accounts for 15–20 % of all breast cancer cases and is characterized by lack of estrogen- and progesterone receptor (ER, PR)-expression as well as lack of human epidermal growth factor receptor-2 (HER2) amplification [1, 2]. Due to the absence of therapeutic targets such as ER, PR or HER2, treatment options for this aggressive subtype of breast cancer are currently restricted to chemotherapy. Although a significant number of patients responds well to conventional chemotherapy, TNBC is generally associated with shorter disease-free and overall survival rates compared to other breast cancer subtypes and comprises about 25 % of all breast cancer-related deaths [1, 3–6]. Alternative therapeutic approaches are therefore highly needed, taking into account the different molecular subtypes within the TNBC group.

Among the quite heterogeneous subgroup of TNBC, a subset of predominantly basal-like cancers appears to share molecular characteristics with BRCA1-associated breast cancer, a phenotype recently described as “BRCAness” [2, 7–9]. Indeed, at least 60–70 % of all breast cancers caused by an inherited *BRCA1* germline mutation are diagnosed as TNBC, while inactivation of the second major breast cancer susceptibility gene *BRCA2* is more frequently observed in hormone receptor-positive breast cancers [10, 11]. Nevertheless, most of the TNBC patients are presenting with sporadic breast cancer and only 9–15 % of all patients within the TNBC subgroup were reported to possess a *BRCA1* mutation [10, 12]. Hence, apart from germline or somatic *BRCA1* mutations, *BRCA1* hypermethylation [12–15] and/or loss of heterozygosity (LOH) [16, 17] may give rise to a BRCA1-like molecular profile in TNBC. Furthermore, Weigman et al. [18] demonstrated frequent loss of several other genes involved in BRCA1-dependent homologous recombination repair in basal-like/triple-negative cancer, most likely contributing to BRCA1-like features. Due to alternative treatment options, information about the BRCA1-like status may have important clinical implications: Various studies have shown that deficiency in homologous recombination (HR) sensitizes the respective tumors to DNA-damaging agents such as platinum compounds [19–22], or to poly(ADP-ribose)polymerase (PARP) inhibitors [23–25]. Accordingly, biomarkers to identify and select patients with BRCA1-like signatures are urgently required.

Based on array comparative genomic hybridization (CGH), we have previously established a BRCA1-like classifier which was highly predictive for the presence of typical BRCA1-associated genomic patterns in breast cancer [26]. Moreover, the arrayCGH-derived BRCA1-like profile proved to be a clinical predictive marker for benefit from high dose platinum-containing chemotherapy [22]. Since the arrayCGH technique cannot be easily implemented in clinical routines, we subsequently translated this rather complex method to a quantitative copy number assay targeting the most specific *BRCA1*-associated genomic regions (3q22-27, 5q12-14, 6p23-22, 12p13, 12q21-23, 13q31-34) by multiplex-ligation-dependent probe amplification (MLPA). The BRCA1-like phenotype, also referred to as “BRCAness”, was defined by applying the previously established shrunken centroid algorithm [26]. In a first study at The Netherlands Cancer Institute (NKI), Amsterdam, Netherlands, the MLPA-based “BRCA1-like test” was able to accurately predict BRCA1-like signatures with 85 % sensitivity and 87 % specificity when compared to arrayCGH as the reference method [27].

In order to evaluate its applicability across a wider range of institutes and countries, we are presenting here an independent validation of the MLPA-based test. The assay was performed on a larger cohort of TNBC patients at the Klinikum rechts der Isar, Technische Universität München (TUM), Germany. MLPA data were subsequently sent to the NKI and classified in a blinded manner. Here we show that approximately half of the TNBC sample set displays BRCA1-like characteristics. Moreover, 83 % of the BRCA*1*-mutated and 91 % of the -methylated tumors, respectively, were correctly classified by the MLPA assay confirming the results of the initial MLPA test. We also searched for further specifications associated with a BRCA1-like signature in TNBC.

## Methods

### Patients and tumor specimens

Fresh frozen breast cancer specimens of the TNBC type which had been collected between 1991 and 2006 at the Department of Gynecology and Obstetrics, Klinikum rechts der Isar, TUM, Munich, were retrospectively used for this study. The TNBC tissues had been macrodissected by a pathologist to assure high tumor content. Samples were classified and assessed for HER2 and steroid hormone receptor (ER, PR) expression at the Department of Pathology as previously described [28]. ER and PR status were defined as negative at less or equal to 3/12 immunoreactive score (Remmele’s score, [29]). HER2-negativity was defined as either immunohistochemistry (IHC) score 0 or 1+ or no amplification demonstrated by FISH in equivocal cases (IHC score 2+). Samples diagnosed for breast cancer before 1999 were retrospectively assessed for HER2 status by IHC and FISH.

**Fig. 1:**
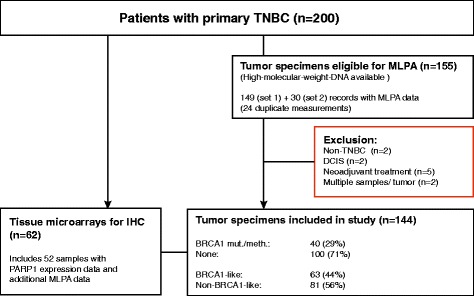
Flow diagram of the study. TNBC, triple-negative breast cancer; DCIS, ductal carcinoma in situ; IHC, immunohistochemistry

For this validation study, 200 unselected cases with documented primary TNBC were included according to availability of fresh frozen tissue-derived material. Out of this patient panel, sufficient amounts of high-molecular-weight DNA could be extracted from 155 samples. A further 9 samples which did not meet inclusion criteria (due to falsely-assigned TNBC subtype, carcinoma in situ, neoadjuvant treatment) were excluded from the final analysis. In cases (*n* = 2) where multiple samples of one tumor were available, only one randomly chosen sample was included (Fig. 1, Flow Diagram). Matched samples which included frozen tumor tissue and paraffin-embedded tissue from the same patient were available for 62 individuals.

### DNA preparation

For DNA preparation, nuclear fractions derived from fresh frozen tumor tissues were used. The nuclear fractions were generated during routine prognostic marker assessment and were obtained by separation from the cytosol preparation by ultracentrifugation [30]. DNA was isolated using the QIAamp DNA Mini Kit (Qiagen, Germany).

### Analysis of *BRCA1* mutations

Detection of small nucleotide alterations within the *BRCA1* coding region was performed by”high resolution melting“(HRM) analysis as previously described [31] using a Lightcycler 480 instrument and the Lightcycler 480 high resolution melting master kit (Roche, Mannheim, Germany). The reaction volume of 20 μl contained 50 ng tumor DNA, 4 mM MgCl_2_ and 10 μl HRM melting master solution. M13 tagged-PCR primer pairs [31] in a final concentration of 250 nM were used. Data analysis was performed with the Gene Scanning module and normalized melting curves were visualized as Difference Plots. Samples indicating differences in melting were subsequently subjected to sequencing analysis on an ABI 3100 capillary sequencer (Applied Biosystems, Darmstadt, Germany). Only clear pathogenic frameshift, nonsense or splice site aberrations were classified as *BRCA1* mutations. International databases such as the BIC database (Breast Cancer Information core: [http://www.research.nhgri.nih.gov]) were searched for these aberrations. *BRCA1* copy number variations in mutation carriers were analysed by the MLPA-based P002-C1 test (MRC-Holland, Amsterdam, The Netherlands) as described previously [32].

### Analysis of *BRCA1* promoter methylation

500 ng DNA was subjected to bisulfite conversion (Epitect Bisulfite Kit, Qiagen, Hilden, Germany) to convert unmethylated cytosin to uracil. *BRCA1* promoter methylation was assessed on a Lightcycler 480-instrument by”methylation-specific high resolution melting” (MS-HRM) analysis employing the Epitect HRM PCR Kit (Qiagen). CpG sites in the studied region were located at position −55 to position +44 relative to the transcription start site at nt 1581 (GenBank sequence #U37574) and covered a transcription-relevant region described earlier by Esteller et al. [15]. Primers are available on request. No relevant amplification of *BRCA1* pseudogene was observed. In brief, 3 μl DNA of the bisulfite reaction was amplified in a reaction volume of 25 μl including 1 μl of each primer (10 μM) and 12.5 μl HRM EpiTect Master Mix. PCR and melting procedures were performed according to the EpiTect HRM protocol (Qiagen) for the Lightcycler 480-instrument.

Normalized melting curves of the tumor DNA samples were compared with serial dilutions of fully methylated and unmethylated control DNA (Qiagen). In concordance with the studies of Lips et al. [27], a tumor sample was assigned as methylation-positive at a degree of ≥20 % methylated sequence. The HRM results were confirmed on a series of five samples by cloning of amplicons (TOPO-TA cloning kit, Invitrogen, Hamburg, Germany) and bisulfite sequencing of 20 clones per sample as described [33].

### Analysis of the BRCA1-like status by MLPA

MLPA analysis is a PCR-based method to analyse the relative copy number of distinct DNA target sequences. In this study, the MLPA probemix P376-B2 for “BRCA1ness” (MRC-Holland, Amsterdam, The Netherlands) was used which contains 34 probes for BRCA1-associated regions, 2 probes for *BRCA1* and *BRCA2*, respectively, and 10 control probes specific for DNA sequences not associated with breast cancer genes. Version B2 of the probemix contains some minor changes in control probes, in comparison with version B1 (ref. [27], original study). In order to compare our data with the original study, data analysis was restricted to 7 control probes by omitting the probes for regions 21q11, 2p11 and 11p15. The assay was performed according to the standard MLPA protocol as described before [34]. One-hundred fifty-five TNBC samples which provided sufficient amount of high-quality DNA (100 ng DNA) were analyzed at the Department of Gynecology and Obstetrics, TUM. Three to four blood DNA samples received from healthy donors and prepared with the same DNA isolation kit as applied for the TNBC samples, were run together with the tumor samples. For normalization, the relative peak areas for each probe were calculated as fractions of the total sum of peak areas in each sample. Subsequently, the fraction of each peak was divided by the average peak fractions of the corresponding probe in the control samples. Relative quantities were finally transferred to an excel sheet and sent to the NKI, Amsterdam, for BRCA1-like class prediction. 144 TNBC samples meeting our inclusion criteria (see Flow chart, Fig. 1) were included for further data analysis. In case of duplicate measurements, only the first experiment was considered.

BRCA1-like class prediction was carried out at the NKI, Amsterdam, using prediction analysis for microarrays (PAM) and R statistics as described before [27]. For the MLPA classifier the cut-off value to classify a sample as ‘BRCA1-like’ was set at ≥0.5. Below this score, a sample was classified as ‘non-BRCA1-like’. The NKI was not aware of the *BRCA1* mutation and methylation status in the TNBC cohort.

### Immunohistochemistry

PARP1 protein expression was measured by immunohistochemistry (IHC) using tissue microarrays (TMA) [28]. TMA sections were deparaffinized and rehydrated through a graded ethanol series finishing with distilled water. Endogenous peroxidase was inhibited by treatment with 3 % hydrogen peroxide. Mouse anti-human PARP antiserum was purchased from BD Pharmingen (catalogue number 551024, clone 7D3-6; San Diego, USA) and applied in a dilution of 1:1500 [35]. Staining was performed with the Dako EnVision Detection System (Dako, Hamburg, Germany) which uses a peroxidase-conjugated polymer backbone coupled to secondary antibody molecules, and diaminobenzidine (DAB+) as chromogenic substrate. Nuclei of the cells were finally counterstained with hematoxylin. Cytosolic and nuclear PARP1 staining intensity, respectively, was assessed by a pathologist in 62 specimens and assigned as absent (0), low (1+), moderate (2+) or strong (3+) staining. Positive controls for PARP1 expression were luminal epithelium of normal breast and BT474 breast cancer cells. Furthermore, additional mammary tissue sections were included in each run as negative controls by omission of primary antibody [36].

Immune cell infiltration was estimated in 53 TMA sections by assessment of CD3 antigen. Staining was performed with the mouse monoclonal antibody MRQ-39 (Cell Marque, Rocklin, CA). Following deparaffinization, antigen retrieval was performed by incubation for 30 min at 95 °C, pH 8.4. Primary antibodies (CD3 1:500) were incubated for 30 min at RT followed by detection of primary antibody using the UV HRP UNIV MULT and UV DAB Kits (Ventana, Tucson, AZ) and counterstaining with hematoxylin. The percentage of positive cells was assessed and classified as no infiltration (0), low numbers of positive cells (1+) and high numbers of positive cells (2+).

### Statistics

Statistical analysis was performed with the IBM SPSS Statistics version 19.0 (SPSS Inc.). Associations between genetic and categorical clinical data were assessed by the Chi-square test. All statistical tests were conducted two-sided and a *p*-value <0.05 was considered indicative for statistical significance. This study was designed according to the REporting recommendations for tumor MARKer prognostic studies (REMARK) guidelines [37]. Data are available on request.

## Results

### Validation of the MLPA-based BRCA1-like test

The validation set contained 144 breast cancer patients with triple-negative subtype. In this patient set, 18 tumors had a germline or somatic *BRCA1* mutation (Table 1), 22 additional specimens exhibited positive *BRCA1* promoter methylation. The MLPA assay initially classified 63 (44 %) tumor specimens as BRCA1-like. We next evaluated whether all *BRCA1*-aberrant tumors had been correctly classified. As illustrated in Table 2, the presence of a *BRCA1* mutation or promoter methylation was predicted with a sensitivity of 83 and 91 %, respectively.

**Table 1:** *BRCA1* mutations in 140 TNBC specimens

Sample	Exon	Nucleotide position (BIC nomenclature^a^)	Effect	Age	Family history of cancer
1	2	c.185_187delAG	fs39X	<50 y	Yes
2	5	c.300 T > G	p.C61G	>50 y	n.a.
3	5	c.300 T > G	p.C61G	<50 y	n.a.
4	5	c.331 + 1G > T	Splice defect	>50 y	n.a
5	7	c.560 + 1delG^b^	Splice defect	>50 y	No
6	11	c.2035 T > A	p.L639X	>50 y	Yes
7	11	c.3600del11	fs1163X	<50 y	n.a.
8	11	c.3600del11	fs1163X	<50 y	Yes
9	11	c.3600del11	fs1163X	>50 y	No
10	11	c.3819del5	fs1242X	<50 y	Yes
11	11	c.3875del4	fs1262X	<50 y	n.a.
12	11	c.3875del4	fs1262X	>50 y	n.a.
13	16	c.5007G > T^b^	p.E1630X	<50 y	n.a.
14	19	c.5298A > T	p.K1727X	<50 y	Yes
15	20	c.5370C > T	p.R1751X	<50 y	Proven somatic^c^
16	20	c.5385–5386insC	fs1829X	<50 y	n.a.
17	20	c.5385–5386insC	fs1829X	>50 y	n.a
18	21	IVS21 + 1G > T	Splice defect	<50 y	n.a.

^a^BIC, Breast Cancer Information core:[http://research.nhgri.nih.gov/bic/]; all variants with the exception of two cases are known pathogenic mutations listed in the BIC database

^b^not found in public data bases

^c^ blood test negative; n.a., not available

**Table 2:** Sensitivity of the MLPA test

	*BRCA1* mutation	*BRCA1* methylation	*BRCA1* mutation/methylation
Total	18	22	40
BRCA1-like (≥0.5)	15	20	35
False negative	3	2	5
Sensitivity (%)	83	91	87.5

BRCA1-like classification with cut-off value ≥ 0.5, non-BRCA1-like classification with cut-off value < 0.5

We looked in more detail onto the false negative data (Table 3). Three misclassified samples carrying a *BRCA1* mutation showed clear heterozygosity at the mutation site and indicated only marginal copy number alterations within the entire *BRCA1* gene (P002-C1 BRCA1 probemix). Moreover, the mutations L639X and K1727X were associated with a distinct phenotype which may indeed reflect the expression of a non-BRCA1-like profile: The L639X-related tumor exhibited a ductulo-lobular-like phenotype and only borderline ER negativity (3/12 immunoreactive score). Similarly, the carrier of the *BRCA1* mutation K1727X had received endocrine therapy reflecting rather ER positivity. Two further discordant samples did not show conspicuous histopathological features, but displayed a BRCA1-like parameter close to the cut-off score 0.5. For one of them, showing positive *BRCA1* methylation, high T-lymphocyte infiltration could be assessed because a matched tumor section of the same patient was available. Thus, normal cell contamination might be a source of misclassification in some samples with values close to the cut-off. We estimated the number of TNBCs with high T-lymphocyte infiltration to up to 38 % using CD3-antigen assessment. However, no relevant association between high immune cell infiltration and a non-BRCA1-like profile was evident in the studied sample set (*n* = 53; Table 4). In addition, only seven of 144 (4.9 %) samples exhibited PAM-R values close to the cut-off score (0.45–0.55) demonstrating that a relative small number of cases would be candidates for repeat analysis. Finally, a further tumor with medullary characteristics might have been misclassified as non-BRCA1-like due to its content of methylated DNA near the applied threshold value (20 %) and/or due to normal cell contamination as well.

**Table 3:** False negative *BRCA1*-aberrant samples

False negatives	Phenotype	BRCA1-like parameter
*BRCA1* mutation		
K1727X	Invasive ductal, borderline ER-negativity, BRCA1 copy number 71 % of normal control	0,18
L639X	Ductulo-lobular, borderline ER-negativity, BRCA1 copy number 82 % of normal control	0,21
fs1829X	Invasive ductal, BRCA1 copy number 85 % of normal control	0,48
*BRCA1* methylation		
20 %	Medullary	0,30
30 %	Invasive ductal, high CD3 counts (2+)	0,499

Cut-off for BRCA1-like parameter: ≥ 0.5; cut-off for positive methylation: ≥20 %

BRCA1 variants are pathogenic mutations with familial background. ER immunoreactivity was classified by Remmele’ score [29]; Loss of heterozygocity (LOH) was analysed by mean copy number loss of BRCA1 probes. T-lymphocyte infiltration was determined by anti-CD3 immunohistochemistry

**Table 4:** Association of the BRCA1-like profile with biological parameters

Variable	Valid cases	MLPA data		*p*-value
	(*n*)	BRCA1-like	Non-BRCA1-like	
Total	144	63	81	
*BRCA1* aberrations	140			<0.000001*
Wildtype	100	28	72	
Mutation/methylation	40	35	5	
T-cell assessment (CD3)	53			0.458
0	17	7	10	
1+	16	7	9	
2+	20	12	8	
PARP1 expression	52			0.087
0–2+	38	17	21	
3+	14	10	4	

BRCA1-like classification with cut-off value ≥ 0.5, non-BRCA1-like classification with cut-off value < 0.5

*statistically significant with chi square test

While *BRCA1*-mutated/methylated TNBCs comprised almost a third (29 %) of the patient cohort, we assigned BRCA1-like signatures in 44 % of the cases. Thus, the specificity of the test for prediction of *BRCA1* aberrations would be moderate (false positive rate 28 %; Table 4). However, it is most likely that additional gene aberrations related to homologous recombination repair are present in the sample set also contributing to the BRCA1-like phenotype.

### Association of the BRCA1-like profile with PARP1 upregulation

**Fig. 2:**
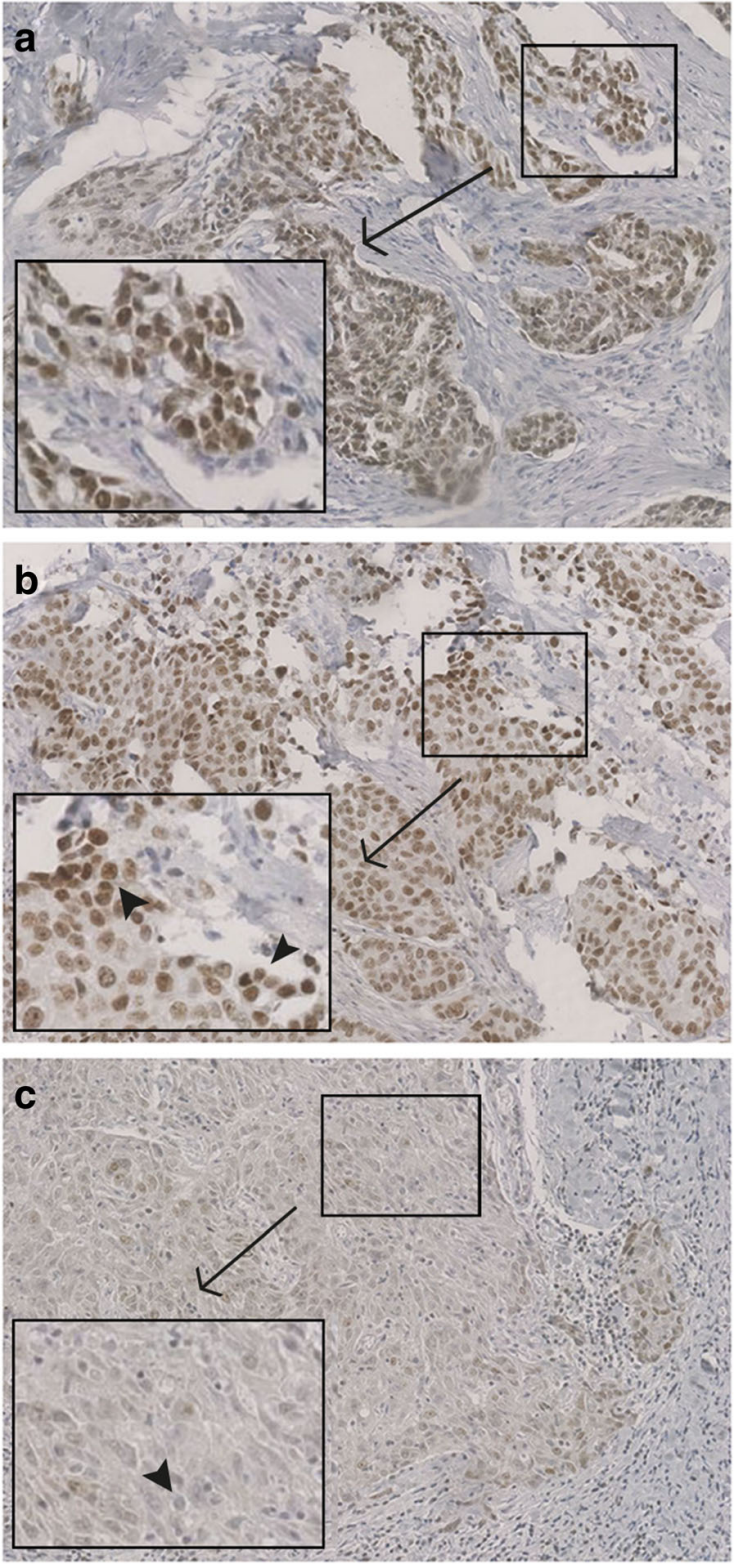
Immunohistochemical PARP1 staining in TNBC tissue microarrays. **a b** BRCA1-like TNBC with high (3+) nuclear PARP1 levels in tumor cells (10× magnification) as assessed by a pathologist. 3+ stained nuclei are exemplarily indicated by black arrows in a separate image section. **c** Non-BRCA1-like TNBC with low cytosolic and nuclear PARP1 levels in tumor cells (10× magnification). Black arrow shows an unstained nucleus. Tissue microarrays were incubated with mouse anti-PARP antiserum followed by staining with peroxidase-conjugated secondary antibody molecules and diaminobenzidine (DAB+) as chromogenic substrate. Nuclear counterstaining was performed with hematoxylin

Since BRCA1-like tumors are supposed to be highly susceptible to PARP inhibitors because of their defects in HR, we evaluated the degree of upregulation of the main target for these inhibitors, PARP1. In a set of 62 matched tumor tissues, nuclear PARP1 protein levels were observed in a range of low (0–1+; 37 %), moderate (2+; 37 %) and strong (3+; 26 %) expression. Cytoplasmic PARP1 expression was generally lower than nuclear expression with 64.5 % of tumors exhibiting low staining, 29 % of tumors with moderate staining and only 6.5 % exhibiting strong staining. The comparison of the degree of nuclear PARP1 expression with BRCA1-like profile revealed a tendency toward higher (3+) PARP1 staining in BRCA1-like vs. non-BRCA1-like tumors (37 % vs. 16 %, *p* = 0.087, *n* = 52) although this was not statistically significant (Table 4 and Fig. 2a–c). A weak, but significant association of high (3+) nuclear PARP1 expression was observed with *BRCA1*-mutated/-methylated cancers compared with wildtype TNBC specimens (50 % vs. 18 %, *p* = 0.016; *n* = 62).

### Association of the BRCA1-like profile with clinical parameters

We next assessed association of the BRCA1-like profile with distinct clinical characteristics of the TNBC patients (Table 5). As expected, BRCA1-like signatures were more prevalent in the group of high-grade (G3) tumors (*p* = 0.0004) and were rarely found in cancers showing histopathological features other than invasive-ductal or medullar (*p* = 0.062). We did not observe association of the BRCA1-like profile with age, nodal involvement or tumor stage. In addition, patients with BRCA1-like cancers had more often received adjuvant treatment (*p* = 0.044) or radiation therapy (*p* = 0.017) compared to the non-BRCA1-like group.

**Table 5:** Association of the BRCA1-like profile with clinical parameters

Variable	Valid cases	MLPA data		*p*-value
	(*n*)	BRCA1-like	Non-BRCA1-like	
Total	144	63	81	
Age	143			0.265
<50	54	27	27	
≥50	89	36	53	
Tumor size	143			0.979
pT 1	54	24	30	
pT 2	69	31	38	
pT 3	8	3	5	
pT 4	12	5	7	
Nodal status	141			0.145
Negative	71	35	36	
Positive	70	26	44	
Histological grade	139			0.0004*
1	6	0	6	
2	28	5	23	
3	105	57	48	
Histology	144			0.062
Invasive-ductal	113	53	60	
Invasive-medullary	11	6	5	
Other	20	4	16	
Adjuvant chemotherapy	142			0.044*
None	34	10	24	
Yes	108	53	55	
Radiation therapy	142			0.017*
None	23	5	18	
Yes	119	58	61	

BRCA1-like classification with cut-off value ≥ 0.5, non-BRCA1-like classification with cut-off value < 0.5

*statistically significant with chi square test

## Discussion

Numerous studies are engaged in the improvement of TNBC outcome, a breast cancer subtype which is still accompanied by unfavorable prognosis [38]. The shared molecular profiles between sporadic TNBCs and BRCA1-associated breast cancer [7, 39], also referred to as BRCAness, may open the way for new therapeutic strategies. In particular, the BRCA1-like profile appears as an excellent molecular marker predicting sensitivity to agents targeting DNA-double-strand-break repair-deficient cancers [25, 40]. Indeed, we could recently demonstrate that BRCA1-like TNBCs show markedly improved outcome after intensified chemotherapy combining alkylating agents such as cyclophosphamide with carboplatin [27, 41, 42]. Most importantly, non-BRCA1-like tumors did not benefit from high-dose alkylating chemotherapy. These observations highlight the clinical relevance of discriminating between BRCA1-like and non-BRCA1-like phenotypes.

A clinically practicable test to identify BRCAness should be robust and easy to implement in routine laboratories. Therefore, we have recently established an MLPA-based assay transcribing the methodology of our former arrayCGH-derived BRCA1-like test into a PCR-based approach [27]. The test proved to be equal to the arrayCGH assay in predicting response to platinum-based alkylating chemotherapy [27]. Our next intention was to confirm robustness and sensitivity of the MLPA-based test across independent laboratories which would be prerequisites for its general application in the clinical setting.

Here we describe a blinded validation of the MLPA test with respect to its ability to predict *BRCA1*-mutated or -methylated samples in an independent cohort of 144 TNBC patients. These were enrolled according to availability of fresh frozen tumor material (nuclear fractions) and amount of high-quality DNA. Clinical properties of the studied patient panel were in concordance with an unselected TNBC patient cohort (see Table 5) although a selection bias cannot be fully ruled out. Speaking against an influence of the selection procedure on the study, the validation test showed very similar sensitivity values compared to our initial results with 87.5 % versus 85 % [27] sensitivity for correct class prediction. In total, five samples could not be correctly classified. We characterized these tumor specimens in more detail: As observed in two *BRCA1*-mutated false negative samples, the presence of hormone receptors and/or ductulo-lobular features might interfere with the expression of a BRCA1-like profile reflected by retention of a wildtype *BRCA1* allele in the analysed tumor section. In this context, we indeed observed that BRCA1-like cancers exhibited more often invasive ductal or medullary characteristics relative to other histological features (see Table 5). Thus, not all *BRCA1*-mutated tumors may generate a BRCA1-like profile probably due to a different etiology or heterogeneity of the tumor.

A second cause of misclassification may be due to normal-cell contamination giving rise to PAM-R values near the cut-off value of 0.5 or below. While low tumor content can be bypassed by microdissecting FFPE samples which are performing equally well in the MLPA test [27], high lymphocyte infiltration would persist. Indeed, Massink et al. [43] reported that the presence of high numbers of tumor infiltrating lymphocytes severely affects tumor profiling, particularly for basal-like, and thus BRCA1-like tumors. We show here that 30–40 % of the TNBC samples (within a subset of 53 samples) exhibited high (2+) T-cell infiltration. Nevertheless, CD3-positive cells were not more abundant in the non-BRCA1-like subset of TNBCs speaking against a major impact of immune cell infiltration on the test results. The sensitivity of the MLPA test might be enhanced in combination with *BRCA1* methylation testing. The methylation assay can also be performed with low tumor cell percentages (minimum 20 %), so nearly all samples will be suitable. In the samples with a tumor cell percentage of 50 % or above, both the MLPA and methylation assay can be performed. In this way, the result should be more robust, and samples with low tumor cell percentage can also be analysed.

In concordance with recent publications [44, 45] we observed that a large proportion (28 out of 63) of the BRCA1-like tumors was not associated with a *BRCA1* mutation or hypermethylation. So far, it is not exactly clear which aberrations beyond BRCA1 abnormalities will cause a BRCAness signature. Lord and Ashworth, 2016, summarized in their recent review [46] the current knowledge encompassing the concept “BRCAness”. Here they define BRCAness as “a situation in which an HR defect exists in a tumor in the absence of a germline *BRCA1* or *BRCA2* mutation”. Considerable evidence is now available suggesting that loss of one or several key genes involved in HR, among these *ATM*, *CHEK1*/*2, NBN, RAD51* and genes of the Fanconi Anemia complementation group, is associated with sensitivity of cancers to platinum drugs and PARP inhibitors. However, an even larger list of HR-modulating genes may also provoke a BRCAness phenotype [46]. Various surrogate measurements for HR defects in cancer such as telomeric allelic imbalance analysis, large scale transition analysis or HRD profiling revealed distinct genomic scars which could be discriminated from confounding alterations not derived from HR deficiency [47]. By performing genome wide expression studies and next generation sequencing, Severson et al. [45] could assign specific gene signatures to the MLPA-derived BRCA1-like profile. They found that genes/pathways involved in DNA recombination, DNA repair and cell cycle were significantly up-regulated. In particular, overexpression of a key regulator of cell cycle progression, FOXM1, and its interactive network may facilitate re-entry of BRCA1-like TNBCs into the cell cycle after DNA damage. FOXM1 was recently found to cooperate with BRG1, a component of the SWI/SNF chromatin remodeling complex, in cellular stress situations [48]. BRG1 is thought to facilitate repair of DNA lesions, e.g. by chromatin relaxation, and was also shown to associate with BRCA1 [49]. Interestingly, the SWI/SNF chromatin remodeling enzymes BRG1 and BRM are mostly overexpressed in breast cancer and their knockout resulted in loss of viability of TNBC cells [50, 51]. Thus, these findings suggest that SWI/SNF components might emerge as potential targets for therapeutic intervention [51–53].

Given that BRCA1-like cells are deficient in HR, PARP1, a key player in base excision repair, may present another selective target for the treatment of TNBC patients. So far, PARP inhibitors have proven to be most effective in *BRCA*-associated familial breast cancers [23–25]. Ossovskaya et al. [54] reported elevated levels of PARP1 mRNA and protein also in TNBC tumor tissues suggesting that TNBC patients might as well be suited for treatment with PARP inhibitors. In the present study, we were interested in the question, whether the BRCA1-like profile might be specifically related to upregulation of PARP1. Indeed we could demonstrate that strong (3+) PARP1 staining was more frequent in BRCA1-like than in non-BRCA1-like tumors. Therefore, at least a subset of BRCA1-like tumors might respond well to the promising treatment option with PARP inhibitors (e.g. in combination with carboplatin).

Interestingly, a recent study observed sensitization of BRCA-proficient TNBCs to PARP inhibitors by inhibition of the PI3K signalling pathway. PI3K blockage resulted in *BRCA1/2* downregulation and impairment of HR [55, 56]. In line with these observations, Severson et al. [45] showed a high frequency of PIK3CA mutations in non-BRCA1-like tumors suggesting susceptibility to PI3K/AKT/mTOR inhibition. Accordingly, these findings would provide a rationale for specific treatment of non-BRCA1-like TNBCs by blocking both PARP1 and PI3K.

## Conclusions

Approximately half of all TNBCs exhibit BRCA1-like characteristics. The BRCA1-like MLPA assay is a fast, simple and cost-effective method suitable for clinical applications to discriminate between BRCA1-like and non-BRCA1-like TNBCs. Moreover, reproducible results were obtained between this study and the initial introduction of the MLPA test. These observations make it particularly attractive compared with other more complex techniques based on genomic scarring. A limitation of this test might be the requirement of high DNA quality and high tumor content. Following the validation of the MLPA-based assay it will now be possible to perform prospective studies which are highly warranted to evaluate the test in a larger setting for predicting treatment benefit from platinum drugs or PARP inhibitors.
